# Effects of ozone for treating chronically refractory wounds and ulcers: A protocol for systematic review and meta-analysis of randomized clinical trials

**DOI:** 10.1097/MD.0000000000020457

**Published:** 2020-05-29

**Authors:** Qing Wen, Dongying Liu, Xian Wang, Yanli Zhang, Song Fang, Xianliang Qiu, Qiu Chen

**Affiliations:** Department of Endocrinology, Hospital of Chengdu University of Traditional Chinese Medicine, Chengdu P. R. China.

**Keywords:** chronic, meta-analysis, ozone therapy, protocol, systematic review, ulcers, wound

## Abstract

**Background:**

The prevalence of chronically refractory wounds and ulcers is growing rapidly. However, the treatment options are not completely effective. Ozone has been demonstrated as being useful in promoting wound healing as well as adverse events in individual studies. Consequently, it is necessary to conduct a meta-analysis of high-quality trials to find out whether ozone therapy is effective and safe in these chronic wounds.

**Methods:**

We will search the Cochrane Library, PubMed, the Web of Science, Embase, CBM, and the Chinese Clinical Registry website without restriction on language, date, or study setting. Randomized controlled trials of ozone therapy for chronical wounds or ulcers will be retrieved in diverse databases from inception to May 2020. The primary outcome of the meta-analysis is the proportion of participants with completely healed wounds; time to achieve complete ulcer healing; change in wound size. The secondary outcomes include the incidence of adverse events, amputation, quality of life, length of hospital stay, and cost. Two reviewers will adopt the Cochrane Collaboration's risk of bias tool to assess the randomized controlled trials and all relevant data will be analyzed by utilizing the Review Manager software V5.3.0.

**Results:**

This study will offer a high-quality synthesis of the effectiveness and safety of ozone for treating chronically refractory wounds and ulcers.

**Conclusion:**

This systematic review and meta-analysis will find out the available evidence to assess whether ozone therapy is beneficial to wound healing and side effects, producing evidence reference for clinical practice on the treatment of wound care.

## 1 Introduction

### 1.1 Description of the condition

Chronical wounds, often known as manifested any breach in the cutaneous continuity, need the length of time to heal more than 3 months, even does not heal and palindromia.^[1,2]^ Wound healing generally conforms to an orderly and timely reparative process following these primary phases of inflammation, angiogenesis, matrix deposition, wound contraction, epithelialization, and cicatrices generation with an appropriate healing time based on various detriment.^[3,4]^ While chronically refractory wounds characterized by interruption of typical progression to healing and delayed rehabilitation are incurred by fibrotic tissue, dead necrotic slough, and multiple infections.^[5]^ A number of causative pathologies of chronical wounds are responsible for different definitions of wound types.^[6]^ It, consequently, would seem that treatment modalities for these encountered commonly causes covering vascular insufficiency, rheumatoid arthritis, diabetes, tumors, chronic osteomyelitis, trauma, burns, hematologic diseases, vasculitis, infection, pressure, or edema are various.^[7,8]^ Only leg ulcers have been reported to impact about 0.45% to 3.33% of population worldwide.^[9]^ The aged people and community with multiple diseases tend to be vulnerable to suffer from this nonhealing wounds and medical resources costs in wound treatment have been exceeded GBP (Great Britain Pound) 1 billion year in the United Kingdom.^[10,11]^

### 1.2 Description of the intervention

Ozone, a gas composed of 3 atoms of oxygen with a cyclic structure, was initially discovered as an oxidant and a disinfectant in 1834 exerting medical effectivity firstly for gangrene during the First World War.^[12]^ Evidence supports ozone has been used for the treatment of cutaneous wounds, including ulcers, with the satisfactory improvement of healing results.^[13]^ Ozone as an auxiliary or alternative method mainly takes the forms of topical performance resorting to ozonized olive or sunflower oil, mixture of zone and oxygen mediated by compresses, tent, bag, even injection, and systematic application referring to rectal insufflation (conveyed into the final portion of the gut/intestines) as well as autohemotherapy (blood withdrawn from body is mixed with combination of oxygen-ozone, then reinfused into the donor).^[14,15]^

### 1.3 How the intervention might work

At present, there is no precision mechanism through which ozone promotes wound healing. The re-establish effects may be accomplished through these hypotheses. Ozone therapy possibly achieves the wound healing via potential induction of growth factors, such as vascular endothelial growth factor, transforming growth factor-β, and platelet-derived growth factor.^[16,17]^ It has been reported the ephemeral and moderate oxidative stress has been generated by ozone acting with the aqueous part of plasma and polyunsaturated fatty acid in vivo, forming reactive oxygen species.^[18]^ A certain amount of reactive oxygen species can act as significant physiological mediators for adjustment by working as vasodilators and that can be beneficial for hemorheology.^[19]^ This multifaced endogenous cascade of responses simultaneously activates the antioxidant system and superoxide dismutase to inhibit oxidative disruption to cellular components.^[20]^ In addition, ozone is able to oxidize the lipoproteins and phospholipids of the membrane to destroy bacteria.^[21]^ In summary, regulation of endogenous growth factors and antioxidant capacity, hemorheology modulations, and pathogen inactivation may exert a comprehensive effect on wound recovery.

### 1.4 Why is important to do this review?

Further research for treatment regimens of chronical wounds that are both clinically valid and cost-effective is warranted due to financial burdens. Alarms have sounded for ozone's toxicity to respiratory passage, skin irritation including dermatitis and burning sensation in the process of administration.^[22]^ Generally, it is unclear whether ozone therapy is effective in these chronically refractory wounds with powerful evidence. Before 2 years, there is only one systematic review about ozone in the treatment of chronical wounds with many deficiencies in this review.^[19]^ Firstly, the database of the literature search is not comprehensive so that some relatively original studies in the review were omitted. Secondly, only the topical ozone application was included without any systematic utilization of zone in study selection. It is doubtable about the summarized conclusion. Therefore, high-quality research is extremely necessary.

### 1.5 Objectives

To find out whether ozone therapy is effective in either of all these chronically refractory wounds; which types of wounds were most suitable, and if those treated experienced any adverse events; whether it is superior to antibiotics for infected wounds.

## 2 Methods

### 2.1 Protocol registration

This protocol has been registered on INPLASY website, and the systematic review protocol will be conducted and reported strictly following the guidelines of the Cochrane Handbook for Systematic Reviews of Interventions and the Preferred Reporting Items for Systematic Reviews and Meta-analysis Protocol (PRISM-P).^[23]^ We will document and illuminate the details in the final report when the critical protocol differences exist in the process of study.

### 2.2 Criteria for considering studies for this review

#### 2.2.1 Types of studies

Only randomized controlled trials on people will be considered for inclusion, irrespective of publication status or language. Cluster-randomized clinical trials and other study designs such as quasi-randomized studies, cohort studies or case–control studies will be excluded as introducing bias due to the cluster effect and obvious heterogenicity by trials’ methods.

#### 2.2.2 Types of participants

Included human participants were of any age with refractory wounds, including war wounds, burns, nonhealing diabetic foot ulcers, venous, or arterial ulcers and cutaneous ulcers of any etiology whether clinically infected or uninfected in any care setting. However, ulcers or wounds in dentistry, palatal epithelial fields, jaw, lung, disc, atmosphere, and meteorology are excluded.

#### 2.2.3 Types of interventions

The primary intervention will be any formulation of ozone topically or systematically applied by any means, alone or in combination with other dressings or components. The comparator group was various involving sham ozone therapy, with concomitant interventions such as antibiotics, topical agents, or conventional care as long as the same concomitant treatment was carried out in both groups; OR any standard treatment regimens designed to promote wound healing.

#### 2.2.4 Types of outcome measures

Primary outcomes:

- Proportion of participants with completely healed wounds (as defined by study authors)
- Time to achieve complete ulcer healing (as defined by authors)
- Change in wound size (as defined by authors)

Secondary outcomes:

- Incidence of adverse events (such as toxicity, irritation)
- Amputation
- Quality of life
- Length of hospital stay
- Cost

### 2.3 Search methods for identification of studies

#### 2.3.1 Electronic searches

We will identify reports of relevant randomized clinical trials via searching the following electronic databases:

- The Cochrane Central Register of Controlled Trials (CENTRAL) (The Cochrane Library) (latest issue)
- PubMed (1947 to present)
- Ovid Embase (1975 to present)
- Web of Science (1946 to present)
- Chinese Biomedical Literature Database (1975 to present)
- The Chinese Clinical Registry (June 2007 to present)
- We will use heading terms plus free words as search strategy which decided by all the reviewers and adopt the following search strategy in the Cochrane Central
- Register of Controlled Trials (CENTRAL):
  #1 MeSH descriptor: [Chronic Disease] explode all trees
  #2 MeSH descriptor: [Wound Healing] explode all trees
  #3 #1 and #2
  #4 MeSH descriptor: [Skin Ulcer] explode all trees
  #5 MeSH descriptor: [Diabetic Foot] explode all trees
  #6 MeSH descriptor: [Leg Ulcer] explode all trees
  #7 MeSH descriptor: [Foot Ulcer] explode all trees
  #8 MeSH descriptor: [Pressure Ulcer] explode all trees
  #9 MeSH descriptor: Burns explode all trees
  #10 MeSH descriptor: [Wound Infection] explode all trees
  #11 MeSH descriptor: [Wounds, Penetrating] explode all trees
  #12 (chronic next wound∗)
  #13 (chronic near ulcer∗)
  #14 ((plantar or diabetic or heel∗ or foot or feet or ischemic or ischemic or venous or varicose or stasis or arterial or skin or leg or mixed or tropical or rheumatoid or sickle cell) NEAR/5 (wound∗ or ulcer∗))
  #15 (bed next sore∗) or (pressure next sore∗) or (pressure next ulcer∗) or (decubitus next ulcer∗)
  #16 (burn or burns or burned or scald∗ or thermal injur∗)
  #17 (wound∗ NEAR/5 infection∗)
  #18 (infusion site∗ or donor site∗ or wound site∗ or surgical site∗)
  #19 (skin abscess∗ or skin abcess∗)
  #20 (gunshot or stab or stabbing or stabbed)
  #21 (#3 OR #4 OR #5 OR #6 OR #7 OR #8 OR #9 OR #10 OR #11 OR#12 OR #13 OR #14 OR #15 OR #16 OR #17 OR #18 OR #19 OR #20)
  #22 MeSH descriptor Ozone explode all trees
  #23 (ozon∗)
  #24 (#22 OR #23)
  #25 #21 AND #24

The search strategies with a little adjustment will be also applicable to PubMed, Ovid Embase, and Web of Science. There are no restrictions based on language, date, or study setting in this review.

#### 2.3.2 Search other resources

We will search manually the bibliographies of all identified and relevant publications to identify any further appropriate trials.

### 2.4 Data collection and analysis

#### 2.4.1 Selection of studies

**Figure 1:**
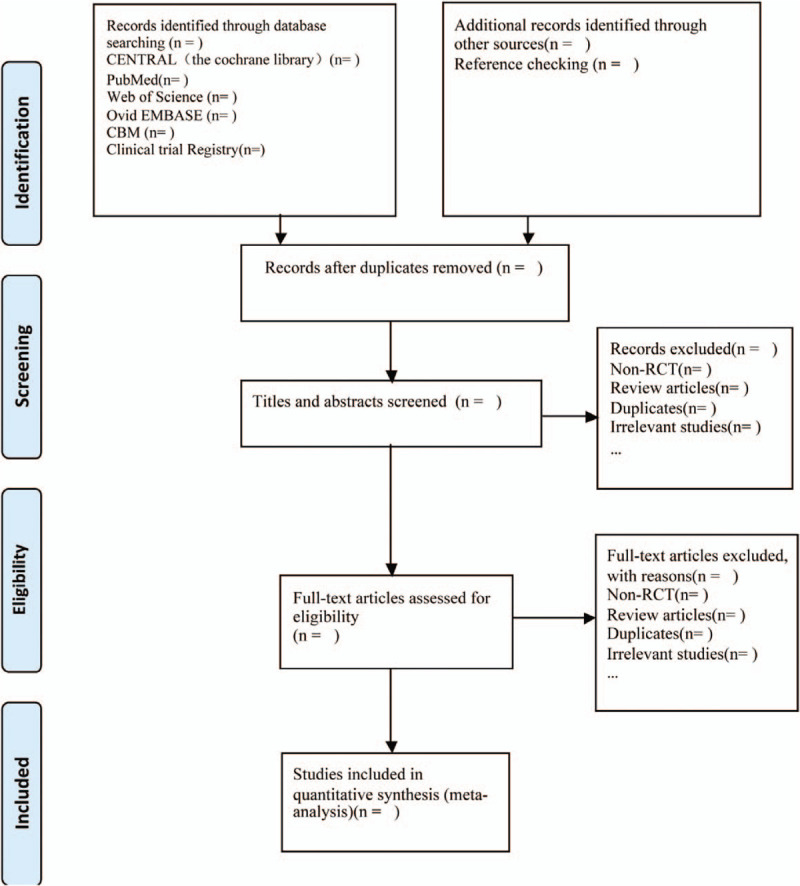
Flow diagram of the study selection. RCT = randomized controlled trial.

We prepare to apply EndNote X8 software to manage all literature. Two reviewers are going to search preliminarily according to our above search strategies by first screening the titles and abstracts of the search results independently. Then full-text copies of all relevant trials will be retrieved for further selection on the basis of inclusion criteria. We will resolve any disagreement through discussion with other review authors until a consensus is reached. Flow diagram about details of the selection will be shown during the screening study (Fig. 1).

#### 2.4.2 Data extraction and management

We are about to use a predefined electronic data extraction spreadsheet for collecting data accordant to the study's requirement. Eligible data will be extracted and recorded by 2 review authors followed by checking carefully via another reviewer. And differences will be resolved by discussion. A reviewer will seek clarification for insufficient or ambiguous information by contacting the authors with e-mails for exact data. The data extracted included the following:

- First author/title
- Year of publication
- Study design
- Location of the trial
- Sample size
- Age and gender of participants
- Wound type
- Intervention and comparison including any concomitant treatments
- Outcomes including methods used to measure outcomes
- Duration of follow-up
- Withdrawals and reason for withdrawal
- Number of participants completing
- Adverse events

#### 2.4.3 Dealing with missing data

If we fail to gain the missing data by contacting authors, a sensitivity analysis will be executed to probe whether these missing data bring about the impact on the overall results of the meta-analysis.

#### 2.4.4 Assessment of risk of bias in included studies

Two review authors will appraise each included study using the Cochrane Collaboration tool for assessing risk of bias on the following 6 specific domains: random sequence generation, allocation concealment, blinding of participants and personnel, blinding of outcome assessors, incomplete outcome data, selective outcome reporting and other biases, such as extreme baseline imbalance. Risk of bias table of each item classified as “low risk,” “unclear,” or “high risk” for each eligible study will be completed and any disagreement about this can be provided consultation to a 3rd independent reviewer.

#### 2.4.5 Measures of treatment effect

We will provide summaries of the intervention effects for each study by calculating risk ratios (for dichotomous outcomes), standardized mean differences (SMDs), or mean differences (MD) (for continuous outcomes). MD can be adopted when the same scale is utilized across different studies to measure an outcome. If not the same scales, SMD is eligible. In addition, its standard error of mean only obtainable in a study will be transformed into standard deviation using a statistical theorem for calculation. We foresee that there will be a paucity for meta-analysis as a result of the scope of different outcomes measured and various wound type across a small number of existing trials, although where studies have employed the same type of comparator and intervention, besides the same outcome measure. 95% confidence intervals and 2-sided *P* values, furthermore, will be computed for each outcome. With regards to pooling the results, Chi-squared test and *I*^2^ statistic will be taken into account for investigating heterogeneity of included studies and we prepare to apply a random-effects meta-analysis with MD or SMD for continuous outcomes and risk ratio for binary outcomes provided *I*^2^ > 50%, *P* < .05 considered as being indicative of substantial heterogeneity.^[24]^ If *I*^2^ < 50%, *P* > .05, this represents negligible heterogeneity with a fixed-effects model. If a meta-analysis is not available, descriptive summarize of individual findings will be performed instead.

#### 2.4.6 Subgroup analysis

Subgroup analysis will be carried out to find out possible sources of statistical heterogeneity following several aspects: wound type, measure of comparators, duration of follow-up, using that provided in each included study.

#### 2.4.7 Sensitivity analysis

We are going to provide sensitivity analyses according to study quality. Omitting the randomized controlled trials with a high risk of bias one by one was followed by pooling the data and analysis again. We can identify the stability of the results and whether an individual study impacts the overall ones by comparing the difference between the original effects and the reobtained results.

#### 2.4.8 Assessment of reporting biases

Reporting biases and will be performed by funnel plot if there are 10 more studies included in this review, and we plan to apply the Egger test for statistical examination depending on *P* < .05 regarded as publication bias.^[25,26]^

#### 2.4.9 Grading the cumulative evidence

The summary of findings tables for each outcome will be made with the Grading of Recommendations Assessment, Development and Evaluate system (GRADE) for assessing the quality of evidence. We will employ the GRADE profiler 3.2 to divide the quality of evidence as 4 grades including “high,” “moderate,” “low,” and “very low” considering 5 relevant factors (limitation, inaccuracy, inconsistency, indirectness, and publication bias).

## 3 Discussion

Given growing mortality and morbidity of chronic wound, a case reported infection pervasion of a nonhealing wound after intralesional ozone injection existing, we hope to identify the impact of effectiveness and safety of ozone on different wound.^[27]^ The systematic reviews are capable of drawing the most reliable evaluation of interventions after careful analysis with more powerful and less biased than the respective studies involved.^[28]^ In this review, we will completely collect clinical studies of ozone on the treatment of chronically refractory wounds and ulcers by 2 authors at least 2 times and adopt systematic review and meta-analysis from different angles with previous review attempts to seek for updated authentic evidence for clinical practice.

## Author contributions

**Conceptualization:** Qing Wen, Yanli Zhang, Qiu Chen.

**Data curation:** Xian Wang.

**Formal analysis:** Qing Wen, Dongying Liu.

**Funding acquisition:** Qiu Chen.

**Investigation:** Song Fang.

**Methodology:** Qing Wen, Dongying Liu, Qiu Chen.

**Project administration:** Qiu Chen.

**Software:** Qing Wen, Dongying Liu.

**Validation:** Xianliang Qiu.

**Writing – original draft:** Qing Wen.

**Writing – review & editing:** Qiu Chen, Qing Wen, Dongying Liu.
